# Immune and Inflammatory Properties of Megakaryocytes

**DOI:** 10.3390/cells14141053

**Published:** 2025-07-10

**Authors:** Shiv Vardan Singh, Audrey Lucerne, Katya Ravid

**Affiliations:** Department of Medicine, Whitaker Cardiovascular Institute, Chobanian and Avedisian School of Medicine, Boston University, Boston, MA 02118, USA

**Keywords:** bone marrow, megakaryocytes, platelets, immune megakaryocytes, hematopoiesis

## Abstract

Megakaryocytes (MKs), which primarily develop in bone marrow (BM) from hematopoietic stem cells, are critical for platelet production. Beyond their well-established role in thrombopoiesis, MKs have been identified as important for BM niche maintenance, such as by supporting the growth and differentiation of other cell types. Recently, megakaryopoiesis has been reported as yielding divergent subpopulations of MKs, as evidenced by single-cell RNA sequencing of lung, spleen, or BM resident MKs. Interestingly, these subpopulations constitute a significant proportion of “immune MKs” expressing various classical immune markers and capable of phagocytosing pathogens and contributing to antigen presentation. As such, MKs were also found to regulate inflammation, mainly by secreting various cytokines and chemokines to crosstalk with other cell types. The level and functional signature of these “immune MKs” were found to be altered in various pathological conditions, indicative of their purposeful values in health and diseases. In this review, we survey and highlight newly reported functional immune and inflammatory properties of MKs in health and in select pathologies.

## 1. Megakaryocytes: Cellular Origin and Importance

### 1.1. Introducing Megakaryocytes

Megakaryocytes (MKs) are specialized cells that give rise to platelets, which are essential for thrombosis and hemostasis [1]. MKs develop from hematopoietic stem cells (HSCs), residing mostly in bone marrow (BM), prior to giving rise to platelets (reviewed in Machlus et al., 2014 [2]). In BM, MKs are the largest cells (50–100 μm) and the rarest, making up approximately 0.5% of total cellular composition [3]. Following an endomitotic cell cycle, MKs become polyploid through escaping cytokinesis, thereby accumulating a DNA content of up to 128n and undergoing a maturation process [4,5]. Recently, single-cell RNA sequencing (scRNA-seq) analysis of MKs has shown the heterogenous nature and subpopulations of BM MKs based on their transcriptional profile [6,7]. As will be noted below, each subpopulation is identified for distinct functions, including platelet-producing, immune function, and HSC niche-supporting [7,8]. Among the functional aspects of MKs, platelet production has been well studied, while in recent years MKs have been identified for regulating hemostasis, wound healing angiogenesis, and more recently also as regulators of inflammation and innate immunity [9,10,11]. The current review focuses on these two latter properties of MKs.

### 1.2. Models of Megakaryocyte Biogenesis Lead to Different Functions

In the mammalian system, hematopoiesis primarily occurs in the embryonic yolk sac and fetal liver during early development, and thereafter in BM during adulthood [12]. HSCs, from which MKs develop, are of two types, having long-term (LT-HSCs) and short-term (ST-HSCs) subpopulations, of which ST-HSCs have reconstitution ability and give rise to multipotent progenitors (MPPs) [13,14]. In the classical model of hematopoiesis, each mature MK is derived from an HSC that sequentially transit through multipotent progenitor (MPP), common myeloid progenitor (CMP), megakaryocyte–erythroid progenitor (MEP), and megakaryocyte progenitor (MkP) states [15]. In recent studies, MK generation through multiple pathways has been proposed, where the process does not entail transit through multipotent or bipotent MEP stages [16]. The concept of a hierarchical lineage tree and stable discrete HSC populations has been revised where the findings of single-cell omics analyses have unraveled novel HSC subpopulations. Similarities have been found between HSCs and MKs, suggestive of a bifurcation of MK biogenesis pathway [17]. Using global and single-cell genomics data, MK-like subsets of myeloid-biased HSCs have been found to have MK-associated genes (GATA1, EPOR, MPL, and vWF) [18]. Additionally, scRNA-seq data also provided novel insights regarding the different pathways of MK biogenesis, along with distinct functions [18,19]. MKs generated via the direct HSC to MkP differentiation pathway are reported to be supportive of the BM niche, whereas MKs generated via stepwise pathway participate in immune regulation of neighboring cell types [8,20], and both pathways may give rise to platelet-producing MKs. Interestingly, direct differentiation of MK progenitors from HSCs has been found to be activated in some pathological contexts, where increased thrombocytosis and thrombosis have been reported. Herein, stem-like MkPs are activated for rapid MK and platelet production, especially during inflammation and inflammatory stress [21].

## 2. Megakaryocyte Presence in and Beyond the Bone Marrow

MKs were found to be localized in fetal liver, spleen, fetal lung, adult lung, and adult bone marrow [22,23]. Based on large OMICS data and corresponding analyses, functional differences between fetal and adult MKs have been identified [18,24].

### 2.1. Megakaryocytes in Bone Marrow

**Figure 1:**
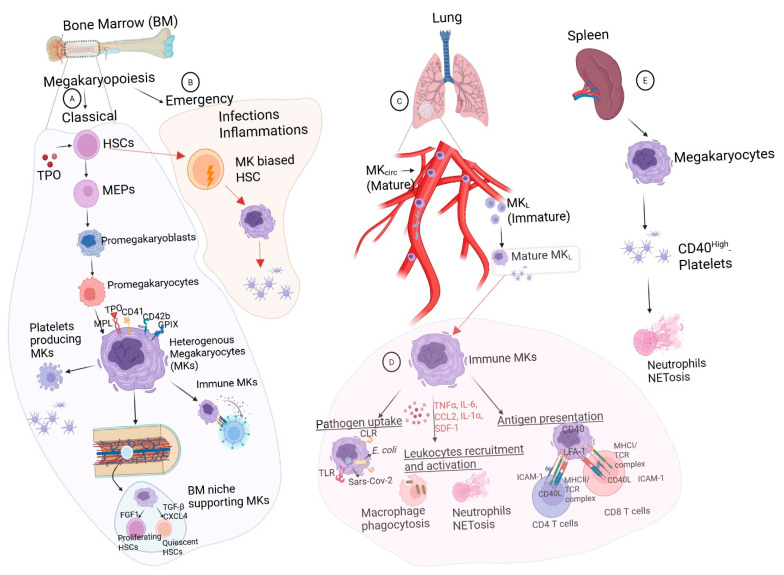
Organ specific megakaryocyte (MK) residents and function. (**A**) MKs participate in maintaining a bone marrow (BM) niche by supporting the growth and differentiation of other cells, including stroma and fibroblasts. MK-derived CXCL4 and TGF-β contribute to maintaining HSC quiescence, whereas FGF1, in response to myeloablative stress, enhances HSC proliferation in BM. (**B**) Additionally, a phenotypic hematopoietic stem cell (HSC) compartment is also reported to have stem-like MK-committed progenitors (SL-MkPs), as a lineage-restricted emergency pool for megakaryopoiesis, especially during inflammatory insults. (**C**) Intravascular (circulatory) lung MKs are reported to produce platelets, which enter the circulation, while extravascular MKs (MK_L_s) are smaller with a typical immune signature. (**D**) MK_L_s are reported to act as antigen-presenting cells (APCs) and activate CD4+ T cells, thus contributing to pathogen recognition and immune responses. (**E**) Spleen harboring immune-skewed MKs were found to produce CD40 ligand^High^-platelets, which also have immunomodulatory functions.

Megakaryopoiesis in the BM microenvironment shares complex physiology comprising an intricate network of endothelial cells (including sinusoids, arterioles, and transition zone vessels), multipotent mesenchymal stromal cells (MSCs) and their progeny (osteoblasts, chondrocytes, and adipocytes), and hematopoietic stem and progenitor cells (HSPCs) and their progeny (MKs and macrophages) [25,26]. Despite representing a minor proportion of cells in BM, MKs with a large size are readily identifiable in the BM [27], where megakaryopoiesis is mainly regulated by thrombopoietin (TPO), which also controls HSC survival and proliferation [28]. In addition to platelet production, MKs play a critical role in the regulation of HSC quiescence by producing transforming growth factor β1 (TGF-β1) and in boosting recovery after stress by secreting transient fibroblast growth factor (FGF), as depicted in Figure 1 [29]. Within a highly vascularized BM, MK migration to the vascular niche has been associated with their platelet generation potential [30]. Based on functional components of BM MKs, four subpopulations have been postulated, (1) active cycling and immature MKs, (2) mature MKs for niche support of HSCs through cell–cell signaling and adhesion, (3) platelet-producing MKs with thrombopoietic phenotype, and (4) MKs having inflammatory properties and playing roles in inflammation [31,32].

### 2.2. Megakaryocytes in Peripheral Blood

The presence of MKs in peripheral blood was initially considered as pathological, such as in the case of myelodysplasia, granulocytic leukemia, or myeloproliferative disorders [33,34]. In the past, studies using peripheral blood smears (PBSs) in normal conditions illustrated MKs with varying shapes (rounded, elongated, etc.), whereas MKs in diseased states may have large cytoplasmic fragments [34]. After the identification of platelet-producing MKs in the lung, the hypothesis of MKs egressing from the BM to the circulation was proposed, since these cells might be filtered out from oxygenated blood by lung capillaries [35]. Additionally, a higher frequency of MKs has been found in blood draining areas rich in BM (such as the cava vein that drains the spine and pelvic bones), compared to forearm veins [36]. This imposes novel insights into the frequency with which MKs can be observed in peripheral blood, depending on the site from which the blood is collected [37]. MKs are rare in circulation and likely fragile and easily overlooked during clinical investigations of blood samples. Additionally, the circulating MK concentration is flexible throughout the lifespan, where aging factors also contribute to altering their count [37]. Based on scRNA-seq data, peripheral blood MKs can be classified into two different clusters: a classical cluster where MKs express typical characteristics and regulate platelets production, with another cluster having pivotal roles in immunity and expressing B cell markers, natural killer cell markers, and T cell markers [38,39]. Overall, investigating peripheral blood MKs in non-pathological or pathological contexts seems to be a promising approach to understanding the role of these cells in distinct locations [37].

### 2.3. Megakaryocytes in the Spleen

Under steady state, rodent spleens are one of the most common sites for extramedullary hematopoiesis (EMH) [40]. Under pathological circumstances, HSPCs migrate out of BM to the spleen, giving rise to MKs. Valet et al. recently isolated splenic MKs for scRNA-seq analysis and found complex immune gene expression of B cell markers, myeloid cell markers, and genes encoding for Fc receptors, MHCII, and immune signals on splenic MKs [41]. Furthermore, these data also revealed that splenic MKs are enriched in immunity-related genes, compared with BM MKs. Ploidy levels in these splenic MKs are widely distributed (8 N–64 N), with a larger proportion of MK-producing platelets having higher expression of CD40L, known for inducing NETosis in neutrophils [42]. This suggests that MKs may have variable functional identities depending on their tissue location and environmental conditions.

### 2.4. Megakaryocytes in the Pulmonary System

Lung MKs were reported in various pulmonary infections, and as a platelet-generating source in thrombocytopenia [43]. In addition, lung MKs were found to be sensitive to various physiological changes, especially inflammation and pulmonary infections. Initially, Howell and Donahue identified in human lung MKs that appeared different in shape and size from MKs in the BM or spleen, suggestive of different functional properties [44]. Other studies of human autopsies and necropsies of lungs are also indicative of a constitutive presence of MKs in the lungs, where their counts are highly variable [45]. Herein, lung MKs are classified into two major categories: (1) circulating MKs (MK_Circ_) and (2) lung resident MKs (MK_L_). Lung MKs were also classified as intravascular and extravascular [46,47]. MKs were shown by electron microscopy in the lung microvasculature with intact, fragmented, and numerous demarcated platelet membranes [47]. Additionally, using intravital imaging in mice, it was shown that at steady state, lung MKs contribute to about 10% of circulating platelets [48], contrary to a much larger and debated percentage (nearly 50%) reported earlier [49]. Transcriptomic analysis of mouse lung resident MKs pointed to enrichment in gene sets related to inflammatory and immune responses, including the Toll-like receptor (Tlr) genes and chemokines [7], as depicted in Figure 1. Ploidy heterogeneity was also found for lung MKs, where low ploidy correlated with an immune phenotype [50]. Moreover, mouse lung MKs displayed an increased expression of MHC II, which enables presentation of ovalbumin peptides to CD4+ T cells, thereby promoting CD4^+^-dependent T cell activation and proliferation. In fact, these “immune MKs” were found to be enriched in the lungs, in accordance with this organ being an interface for environmental stress [51]. In humans, lung MK counts were augmented in various pathological conditions, such as in coagulation-related diseases, tissue damage and bleeding (shock, burns, and hemorrhage), cancer, inflammatory lung diseases, idiopathic pulmonary arterial hypertension, fibrosis, asthma, and viral infections [51,52].

## 3. Immune Identity of Megakaryocytes

MKs express various immune receptors that participate in pathogen recognition and response, phagocytose of pathogens, antigen presentation, and also interaction with other immune cell types.

### 3.1. Megakaryocytes as Immune Cells

Human MKs possess significant immune potential in addition to their primary role of platelet production. MKs sense inflammation, present antigens, and modulate immune responses through cytokine release and antiviral immunity. Upon encountering inflammatory challenges, these cells exhibit intricate immune functions to control inflammation through various mechanisms, such as secretion of anti-inflammatory or pro-inflammatory cytokines and release of immunomodulatory platelets, specific to various pathological conditions, such as myelofibrosis, immune thrombocytopenia, and inflammatory arthritis [46,48]. Human MKs express various immune receptors and molecules associated with immune recognition and response, such as Toll-like receptors (TLRs), Fc gamma receptors (FcγRs), and CD40L [53,54], as further detailed in Table 1. These immune receptors are well known for their association with other immune cells, including monocytes, T and B lymphocytes, and natural killer (NK) cells, thus allowing MKs to recognize pathogen-associated molecular patterns [54,55]. These “immune MKs” and their receptors also interact with pathogen-specific antibodies, hence advocating their antibody-mediated clearance [56]. Human MKs were also reported to release cytokines, such as platelet factor 4 (PF4 or CXCL4), a proliferation-inducing ligand (APRIL), TGF-β, IL-8, CXCL1, IL-1α, IL-1β, and IL-6 to regulate B cell and plasma cell development and survival, and to influence immune cell activity and modulate inflammation [57]. An anti-viral immune gene signature of human MKs was proposed to be of translational value by enhancing host responses to limit viral infection. For example, overexpression of interferon-induced transmembrane protein 3 (IFITM3) in human MKs was found to be significant in limiting viral infection [58].

Similar to human MKs, primary mouse MKs were found to possess significant immune properties, thus participating in both innate and adaptive immune responses. Mouse MKs express various immune sensors like TLRs, Fcγ receptors, dendritic cell markers, and acting as antigen-presenting cells (APCs), thus contributing to immune surveillance and T cell activation. A list of various immune markers identified in mouse MK subpopulations is outlined in Table 2. As identified, mouse primary lung MKs also express phagocytic behavior. Using a fluorescence-mediated live imaging tool in mice, *E. coli* was found to be internalized into phagolysosomes for digestion by BM and lung MKs, where lung MKs have more potential for phagocytosis [59]. In addition to phagocytic behavior, mouse “immune MKs” also possess antigen-presenting potential since MkPs were found to carry complex class II (MHCII) molecules, which are responsible for inciting Th17-driven autoimmunity [60]. The activation of Th17-driven immune response is mainly induced by secretory cytokines such as, interleukin-1 (IL-1), IL-6, TGF-β, and IL-23 [60,61]. Compared to MkPs, murine mature MKs express both MHCI and MHCII, which upon exposure to exogenous antigens effectively triggers the activation and proliferation of CD4+ and CD8+ T cells [62]. Additionally, mouse “immune MKs” express CD40L, which is known to be presented on activated CD4+ T cells to enhance their antigen-presenting potential mainly through B cell maturation and macrophage-directed phagocytosis [57,58]. Remarkably, mice lacking PF4 have delayed maturation of B cell lineage in the BM environment [63,64]. This points to the vitality of “immune MKs” for the proliferation of HSCs, which reside in direct contact with MKs, where the expression and release of PF4 and TGF-β directly regulate HSC quiescence and proliferation [64]. Collectively, as identified in human and mice, MKs possess a substantial immune signature, which drives hematopoietic stem cell maintenance and immune response modulation.

**Table 1:** Megakaryocyte (MK) subpopulations and their specific gene/immune markers with reported functions in humans.

Characteristics	In Bone Marrow	In the Lungs	In Peripheral Circulation	In the Spleen
Functional diversity	Bone marrow (BM) immune MKs regulate inflammatory responses, myeloid leukocyte activation, leukocyte-mediated immunity, and cellular responses to cytokines and interferons [29]	Adult lungs are home to CD42^+^ mature MKs [53,54,55]	Circulating MKs express various markers specific to platelets and to the lineage, such as CD61, CD41, CD42b, and PF4, of which some take part in coagulation and chemotaxis [38,39]	MKs are primarily associated with extramedullary hematopoiesis (EMH). Splenic MKs can interact with various immune cells, including myeloid cells and T cells [41].
Immune gene expression	MKs express immune genes such as, S100A9, IL-1β, TLR2 and TLR4, CTSS, IL1R, IL10R, IFN-γ, HLA-DRA, CD48, and CD148 [55,61]	MKs express MHCII, TLRs, chemokines, and CD74 [53,54,55]	MKs express various immune signaling molecules and receptors, such as S100A8/A9, IL-8, IL-1β, TNFα, TLR2, TLR3, TLR4, ICAM1, and MHCII, as studied in SARS-CoV-2 patients, where these markers were found to be highly upregulated [54,61,65,66]	MKs release cytokines such as TNFα and IL-6, which promote bacterial phagocytosis, and produce immune-functional platelets, which can activate neutrophils and induce NETosis, contributing to microbicidal effects [41,42].

**Table 2:** Megakaryocyte (MK) subpopulations and their specific gene/immune markers with reported functions in mice.

Characteristics	In Bone Marrow	In the Lungs	In Peripheral Circulation	In the Spleen
Functional diversity	BM immune MKs participate in immune and inflammatory responses, wound healing, and platelet activation [67]	Immature, low-ploidy MKs express vital immune markers [45].	MKs participate in innate and adaptive immune responses, antigen processing and presentation, and T cell co-stimulation [38,39,68]	MKs ploidy level is widely distributed (8 N–64 N), and these cells bear immunomodulatory functions, which reduces mortality in a mouse model of sepsis [41]
Immune gene expression	MKs express various immune genes related to leukocyte-mediated immunity and cytokine- and interferon-mediated cellular responses [30], such as *Spi1*, *Cebp*, *Irf*, *CD53*, *Ccl3*, *Lsp1*, *Cxcr4*, *Ccl4*, *Il17r*, and *Cdh1* [69,70]	MKs have high expression of MHCII, TLRs, chemokines, and CD74 [62]. MKs have higher surface expression of immune regulatory molecules responsible for antigen uptake, processing, and presentation to CD4+ T cells, as studied in a lung bacterial infection mouse model [71].	MKs express typical lineage markers, such as CD61, CD41, CD42b, and PF4 [62]	MKs express B cell markers, myeloid cell markers, MHC II and CD40L, which impart NETosis [42]

### 3.2. Megakaryocyte Subpopulations and Their Immune Signature

Recent scRNA-seq data highlighted the presence of MKs subpopulations with immune signatures, both in humans and mice [19]. Interestingly, extramedullary MKs were found to be more immunoregulatory, compared to BM MKs, also evident from various scRNA-seq datasets [18,19]. A study by Wang et al. unveiled a novel landscape of human embryonic MK heterogeneity and delineated the developmental trajectories of early megakaryopoiesis on the basis of droplet-based scRNA-seq data derived from the yolk sac at 4 weeks post-conception and from fetal liver at 8 weeks post-conception [72]. Additionally, during embryonic development, “immune MKs” exhibit enriched expression of genes associated with phagocytosis, antigen processing and presentation, and macrophage-specific (C1QC) genes [73,74]. During fetal development, MKs are characterized with the specific immune marker CD14 (soluble component of TLR4), having a potential role in activating innate immune response [73,75]. In contrast, human adult “immune MKs” display a diverse immune profile with roles in innate and adaptive immunity [76]. This includes pathogen recognition, phagocytosis, cell-mediated killing, antigen presentation, and neutrophil recruitment.

In studies using mice, a modified Smart-seq2 protocol was implemented to characterize low-ploidy and inflammatory-response-associated adult MK subpopulations, showing high expression of immune genes in these low-ploidy cells [50]. These conserved gene signatures also aided in evaluating the immune properties of primary MKs through in vitro regeneration. Interestingly, studies by Qin et al. and Rodríguez et al. used these markers to identify “immune MK” subpopulations during in vitro thrombopoiesis, where cells were derived from human cord blood [77,78]. Mouse BM MK subpopulations with an immune signature (approximately 7% of total MKs) have been identified to be enriched in inflammatory response and myeloid leukocyte activation properties, through expression of leukocyte-specific protein 1 (LSP1) and CD53 [29]. These immune signature genes of BM MKs are associated with leukocyte-mediated immunity and cellular responses to cytokines and interferons. This subpopulation is also characterized by high expression of C-C motif chemokine ligand 3 (*CCL3*), which is a potent activator of innate and adaptive immune responses. Additionally, inflammation-associated genes have been remarkably identified in this subpopulation, such as *S100A11* and *S100A12* (S100 calcium-binding protein A11 and A12) and TNF-α-induced protein 3 (*TNFAIP3*) [54,61]. Mouse lung MK subpopulations have also been identified with distinct immune phenotypes and functions, compared to BM MKs, with the potential to release inflammatory cytokines and molecules, parallel to tissue resident leukocytes and antigen-presenting cells (APCs). The mouse lung MK immunomodulatory phenotype includes higher surface expression of immune-regulatory molecules responsible for antigen uptake, processing, and presentation to CD4^+^ T cells and subsequent CD4^+^ T cell activation, as evident in a lung bacterial infection model [45,47]. scRNA-seq datasets of mouse lung MKs are indicative of phenotypically distinct immune gene expression profiles that are similar to those in dendritic cells and classical antigen-presenting cells. Herein, these lung MKs are capable of presenting OVA-peptide via MHC-II to CD4^+^ T cells both in vitro and in vivo [49]. Finally, splenic MKs, unlike their BM and lung counterparts, were identified for their platelet production subpopulations with higher CD40L expression, which can activate neutrophils and induce NETosis (formation of neutrophil extracellular traps) as part of a microbicidal process [41,42].

Taken together, scRNA-seq data from BM, fetal and adult MKs, and lung MKs show distinct functional profiles of “immune MKs” [19,65]. Collectively, these findings are suggestive of diverse subpopulations of immunogenic MKs in various tissue types, as further summarized in Table 1 and Table 2.

## 4. Immunoregulatory and Inflammatory Functions of Megakaryocytes in Select Diseases

The network of MKs and their identified crosstalk with “immune cells” is highly relevant to disease states [66,79]. This is illustrated here in four different cases outlined below: mutation-induced blood cells dysregulation, viral infection, aging, and sepsis.

### 4.1. In Myeloproliferative Neoplasms

Myeloproliferative neoplasms (MPNs), which constitute a heterogenous group of related hematological disorders and mainly affect cells of myeloid lineages [80,81], are caused by clonal mutations such as *Janus Kinase 2* (JAK2), thrombopoietin receptor (myeloproliferative leukemia protein; *MPL*), and *Calreticulin* (CALR) genes [81,82]. These are centrally linked with TPO over-expression, which induces the JAK/STAT, MAPK/ERK, and PI3K/AKT pathways, resulting in increased MK number and myelofibrosis [82,83]. In addition to TPO, various other secretory components are released by murine MKs to regulate megakaryopoiesis and affect the BM niche, including transforming growth factor-β (TGF-β), C-X-C motif ligand 4 (CXCL4), fibroblast growth factor 1 (FGF1), platelet derived growth factor (PDGF), IL6, oncostatin M, and bone morphogenic proteins (BMPs) [82,83]. A dataset of MPN patient samples showed an elevated level of inflammatory cytokines (IL8 and TGF-β), which are known to promote MK expansion [84]. These secretory components were found to be pivotal in the pathobiology of MPNs. TGF-β is upregulated and secreted by abnormal MKs and monocytes and it directly stimulates the fibroblasts and other cells of the extracellular matrix [85]. CXCL4 promotes fibrosis through an effect on the differentiation of stromal cells [86]. Similarly, the proinflammatory cytokine IL-6 was found to be elevated in MPN patients and in murine models, and neutralizing antibodies specific to IL-6 or its genetic deletion reduced erythropoiesis and myeloproliferation [87].

### 4.2. In Lung Diseases and COVID-19

In coronavirus disease 2019 (COVID-19) patients, a remarkable increase (up to 7–10-fold) in lung and circulating MK count was identified [88]. Lung MKs were found to be augmented in various other diseases such as thromboembolic disease, intravascular coagulation, myocardial infarction, and severe atheroma [88,89]. In COVID-19 non-survivor patients as well as in influenza patients, a higher degree of MK maturation was found in the lung vasculature [90]. Interestingly, pathogenic mechanisms explaining elevated levels of MKs seem to be associated with platelet loss and bleeding, as evidenced by hyper-inflammation and augmented thrombopoietic activity [91]. Compared to BM, lung MKs have higher levels of pattern recognition receptors, such as Toll-like receptors (TLRs) and C-type lectin receptors (CLRs), which enable cells to phagocytose bacteria (*E. coli*) and viruses (SARS-CoV-2; severe acute respiratory syndrome coronavirus 2) [92,93]. Additionally, lung MKs act as potent antigen-presenting cells to modulate T cell response through major histocompatibility complex (MHC) class I and MHC class II molecules, similar to leukocytes and antigen-presenting cells in humans [93]. Accordingly, a recent study reported that lung MKs internalize and process antigenic proteins and bacterial pathogens [94]. Furthermore, lung MKs also induce CD4+ T cell activation in an MHC II-dependent manner, where CXCR4^high^ MKs express ovalbumin surface antigens via MHC I-dependent activation of CD8+ T cells [93,95]. Additionally, upon lipopolysaccharide (LPS) stimulation, mouse lung MKs show higher expression of inflammatory molecules [96]. These include chemokine (C-X-C motif) ligand 1 (CXCL-1), soluble intercellular adhesion molecule 1 (sICAM-1), IL-1α, SDF-1, macrophage inflammatory protein 3 (MIP-3), IL1RA, tumor necrosis factor alpha (TNF-α), and chemokine (C-C motif) ligand 2 (CCL-2) [96,97]. Following infection with influenza or SARS-CoV-2, MKs express a higher level of the *IFITM3* gene, which encodes for interferon-induced transmembrane protein 3 and plays critical role in host immunity against viral infections [58,98]. SARS-CoV-2 infection modulates the transcriptomics of MKs in the lungs, bone marrow, and peripheral circulation [99]. Circulating MKs infected with SARS-CoV-2 release augmented levels of IL-6 and IL-1β cytokines, which is mediated by the nuclear factor κB (NF-κB) pathway [100]. In the lungs, infected MKs release vascular endothelial growth factor (VEGF), platelet-derived growth factor (PDGF), and other inflammatory molecules in BM-derived human adult MSCs [101]. Additionally, lung MKs are enriched with several growth factors (TGF-β1, FGF, SDF1, IGF1, and CLEC1B), known for pulmonary alveolarization, growth, and development [99]. Collectively, viral infections affect MKs properties, resulting in hyperinflammation and contributing to aggravated pathology.

### 4.3. In Aging

Aging has been associated with elevated noradrenergic innervation to promote megakaryopoiesis via a β2-adrenergic receptor and IL6-dependent pathway in BM [102]. Aged mouse HSCs were reported to be MK-biased, with a higher count of mature MKs and platelets, compared to a young mouse population [103]. These changes also induce mitochondrial dysfunction and altered inflammatory pathways in mouse MKs, including upregulation of inflammatory stressors, such as TNFα and mTORC1 signaling [104]. Interestingly, the level of MK-generated PF4 was found to be lower in serum samples of aged mice and humans, where its systemic administration again rejuvenated altered neuroinflammation and cognitive functions. Moreover, MKs were found to be regulatory for BM HSCs differentiation in aged mice, where MKs follow a direct differentiation pathway [105]. These findings suggest that MKs play a pivotal role in systemic aging processes.

### 4.4. In Sepsis

In the pathophysiology of sepsis, coagulation, inflammation, and immunity serve as a trinity of pathophysiological changes, where their mutual influences and network affect the disease progression. Physiological changes in MKs particular to coagulative, inflammatory, and immune functions also impact sepsis, where in early stages and progression of sepsis, patients often experience varying degrees of thrombocytopenia [106,107]. The typical manifestations of sepsis-induced coagulopathy (SIC) include upregulated level of procoagulant factors and downregulated anticoagulant factors, along with impaired fibrinolysis [108]. Platelets and their precursor MKs are activated in sepsis, and express various immune receptors. Herein, TLR2 and TLR4 enable mouse MKs to sense immune signals where the activation of these receptors induces MKs hyper-maturation and platelet overproduction [68,79,109]. In LPS-treated mice, increased levels of TPO and cytokines lead to activation of TLR2 and TLR4 through a PI3K/NF-κB axis in HSPCs and MKs [106,110]. Additionally, angiopoietin-like 4 (ANGPTL4) was found to regulate STAT3 expression in immature mouse MKs during sepsis and also to promote platelet overproduction [111]. Furthermore, extracellular vesicles (EVs) have been found to be of diagnostic and therapeutic values in sepsis, where MKs and platelet-derived EVs participate in thrombogenesis and immune response [112]. Platelet EVs are potent inducers of procoagulant responses in sepsis [112], with potential to activate endothelial cells, promote leukocyte migration, and interact with neutrophils to mediate NETosis [42,112]. This suggests that MK/platelet-derived EVs have regulatory roles in hyperinflammation during sepsis.

## 5. Conclusions and Future Prospectives

MKs have been emerging as cells with novel immune functions of clinical value in various infectious diseases and pathologies, such as COVID-19 and MPN. Studies point to BM MKs as a key source of platelets [113], while debates over the extent of platelet production in the lungs are still ongoing. The present review highlights the developmental origin of “immune MKs” with their localized presence and importance. Breakthroughs in scRNA-seq and emerging transcriptomic datasets have revealed distinct characteristics and immunoregulatory functions of these “immune MK” subpopulations and their signaling crosstalk with conventional immune cells. Yet, variability in scRNA-seq results across platforms needs to be considered prior to drawing firm conclusions. Confirmation at the protein level of key cellular pathways in different MKs is still missing. Immunoregulatory roles of these cells in selected diseases provide novel insights towards possible translational application. Future studies are warranted on proteomic signatures of MKs in the BM or other sites, whether organ residents or circulation, and the role of these cells in various pathologies.

## Data Availability

Not applicable.
